# Crimean-Congo hemorrhagic fever: epidemiological trends and controversies in treatment

**DOI:** 10.1186/1741-7015-9-131

**Published:** 2011-12-08

**Authors:** Helena C Maltezou, Anna Papa

**Affiliations:** 1 Department for Interventions in Health-Care Facilities, Hellenic Center for Disease Control and Prevention, 3-5 Agrafon Street, Athens, 15123 Greece; 2 Department of Microbiology, Medical School, Aristotle University of Thessaloniki, Thessaloniki, Greece

## Abstract

Crimean-Congo hemorrhagic fever (CCHF) virus has the widest geographic range of all tick-borne viruses and is endemic in more than 30 countries in Eurasia and Africa. Over the past decade, new foci have emerged or re-emerged in the Balkans and neighboring areas. Here we discuss the factors influencing CCHF incidence and focus on the main issue of the use of ribavirin for treating this infection. Given the dynamics of CCHF emergence in the past decade, development of new anti-viral drugs and a vaccine is urgently needed to treat and prevent this acute, life-threatening disease.

## Background

Crimean-Congo hemorrhagic fever (CCHF) is an acute, highly-contagious and life-threatening disease caused by a nairovirus of the *Bunyaviridae *family [1-3]. CCHF was probably described by a physician in Tajikistan in 1100 AD in a patient with hemorrhagic manifestations [2]. In recent times, the disease was first recognized during an outbreak in Crimea in 1944, however, later it became evident that the causative agent was identical to a virus isolated from a patient in Congo in 1956, and the name CCHF was adopted [4]. CCHF virus (CCHFV) circulates in nature in a tick-vertebrate-tick cycle, mainly among cattle, sheep, goats, and hares. The infection is transmitted to humans primarily by ticks of the genus *Hyalomma*, but also through direct contact with blood or tissues of viremic patients or animals [1,2]. Typical CCHF progresses rapidly with high fever, malaise, severe headache, and gastrointestinal symptoms. CCHF is confirmed either by detection of specific immunoglobulin M antibodies or a four-fold increase of immunoglobulin G titers using enzyme-linked immunoassays, indirect immunofluorescent assays, or through reverse transcriptase-polymerase chain reaction and microarray techniques [1,5,6]. Prominent hemorrhages may occur at a late stage of disease, with case fatality rates ranging from 5% to 50%. CCHF is a disease of immediate notification to public health authorities because of the potential of nosocomial outbreaks [7-10] and use in bioterrorism [11].

**Figure 1:**
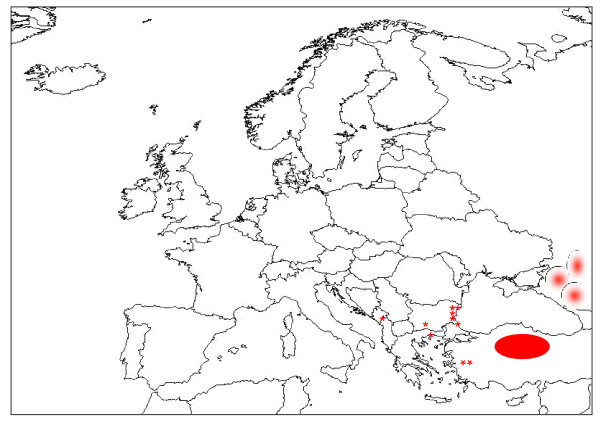
**Foci of CCHF emergence or re-emergence in southeast Europe and neighboring countries from 2000**. Stars indicate sites of CCHF emergence in southeastern Europe; oval indicates the epicenter of the large CCHF epidemic in Northeastern Anatolia, Turkey; and circles indicate the site of CCHF re-emergence in Southwestern Russia (Data from References 12 and 17). The Figure was created based on a map outline available at: http://geography.about.com/library/blank/blxeurope.htm. Abbreviations: CCHF, Crimean-Congo hemorrhagic fever.

CCHFV has the widest geographic range among all tick-borne viruses, being endemic in more than 30 countries in Eurasia and Africa [1,5]. CCHF activity has increased over the past decade and new foci have emerged in several Balkan countries, as well as in neighboring areas (Figure 1) [5,8,12-15]. In a serosurvey conducted in Northeastern Greece after the first human case occurred in June 2008, seroprevalence rates up to 5% were found in well-confined areas compared with 0% found in the same areas 20 years ago [16], suggesting recent introduction of the virus. After almost three decades, CCHF re-emerged in southwest Russia in 1999, with hundreds of cases being reported since then [17]. An essential factor of the CCHF re-emergence in Russia was a rise in the number of *Hyalomma marginatum *ticks [18]. Enzootic circulation of the CCHFV was documented in Turkey for several decades, however the first human cases occurred in 2002; since then, Turkey has experienced the largest ever recorded CCHF outbreak with more than 4,400 confirmed cases, mainly from rural areas in Northeast Anatolia [19]. At the epicenter of this outbreak, the seroprevalence rate was 12.8% among high-risk groups [20]. Recently, 26 cases were identified in western Turkey [21], indicating further spread towards the west in this country, which is attributed to the vegetation fragmentation in this area, as well as in Anatolia, in association with increased awareness of this disease following the 2006 campaign [21]. This article discusses factors influencing CCHF incidence and focuses on the main issue at stake: the use of ribavirin for treatment.

### Factors influencing CCHF incidence

As for all vector-borne diseases, environmental factors, climate, and human behaviour are critical determinants for the establishment and maintenance of CCHF endemicity within an area. However, although explanations about CCHF emergence or re-emergence have been formulated [5,17,22-24], the contribution of each of these factors has not been quantified so far.

Humans may modify the risk of CCHFV transmission through changes in land use, recreational activities, and livestock movement [5], and increasing awareness may also impact the incidence of CCHF [21], whereas limitations in diagnostic capacities and surveillance may hamper the estimation of CCHF burden in several endemic areas [17].

*Hyalomma *ticks favor dry climates and arid-type vegetation, and are abundant in European countries bordering the Mediterranean Sea, along with numerous animals which may act as CCHFV hosts [12]. However, their established presence does not ensure CCHF expansion to these areas *per se*. Besides most Balkan countries, which are well-known to be endemic for the presence of CCHF for several decades, serologic evidence of CCHF is scantily available elsewhere in Europe [12,25,26]. Introduction of CCHFV to a non-endemic area could happen either through legal or illegal trade of infected animals or animals infested with infected ticks or through geographic expansion of infected *Hyalomma *ticks from CCHF-endemic to CCHF-naive areas [27,28].

A model investigating the impact of several climate scenarios on ticks, has found that a rise in temperature and decrease in rainfall in the Mediterranean region could result in a sharp rise of the distribution of suitable habitat for *Hyalomma *ticks northwards, with the highest impact at the boundaries of their current range [29]. CCHFV infected animals only experience viremia for a few days, which is likely an intrinsic limit for the establishment of successive transmission cycles after their importation, beyond the onset of a localized outbreak [12]. CCHFV seroprevalence rates among animals are good predictors of human risk for infection [14]. Given the dynamics of CCHF over the past decade, further studies on ticks and animals will allow the detection of presumed unaffected foci with suitable biotic or abiotic conditions for CCHF emergence [22,23], with the highest risk in neighboring areas to those with established endemicity.

### Control strategies

Given that the control of ticks is an unrealistic goal [30], strategies should focus on enhancing surveillance using standardized case definitions [31], and increasing laboratory capacity within already endemic areas and areas at risk for CCHF expansion [17]. The general population and health-care workers should be aware of prophylactic measures and modify their risk for infection.

An inactivated suckling mouse brain vaccine was developed in the former Soviet Union in the late 1960's with no adverse effects recorded on the limited number of volunteers [30]. Data from approximately 2,000 healthy persons vaccinated in 1970 indicated that neutralizing CCHFV antibodies developed 1 to 4 weeks after the third shot, but titers decreased 3 to 6 months later [30]. The vaccine has been used for high risk groups in Bulgaria since 1974. Although surveillance data suggest a four-fold reduction in the number of notified CCHF cases in Bulgaria after the introduction of vaccination, data regarding vaccine efficacy have not been published [30]. Recently, the CCHFV strain which is used for vaccine preparation was genetically characterized, providing the basis for further studies [32]. Overall, there are concerns about using mouse brain vaccines because of possible autoimmune responses. CCHF is mainly confined to poor resource countries, and research has been extremely slow. A humanized vaccine against CCHF is needed, however long-term in-field studies will be required to show efficacy.

### Issues of treatment and prophylaxis

To date only ribavirin, a broad anti-RNA virus inhibitor, demonstrates *in vitro *activity against CCHFV either in cell culture [33,34], or in mice models [35,36]. In the historical study conducted by Mardani *et al*. in Iran during 1999-2001, the case fatality rate was 11.6% (8 of 69 patients) in the group treated with oral ribavirin compared with 58.3% (7 of 12 patients) in the untreated group, which corresponded to 80% efficacy (*P *< 0.001) [37]. This study constituted the basis for the World Health Organization (WHO) recommendations for treating CCHF patients with ribavirin. Promising results were also reported by others, and were mainly associated with early treatment [38-40]. However, the use of ribavirin for CCHF treatment remains an issue of controversy since no difference in case fatality rates was found in other studies [41-43], including the only randomized controlled trial published on this topic so far (6.3% among 64 ribavirin-treated patients versus 5.6% among 72 untreated patients; *P *= 0.86) [42]. Almost all data about ribavirin efficacy are limited to small observational studies and case series, and questions about methodological issues have been raised (for example no control for disease severity or day of initiation of treatment) [17]. In a recent meta-analysis of 21 studies assessing the efficacy of ribavirin on CCHF outcome, the authors concluded that current evidence is insufficient to provide a clear answer [44]. Currently, ribavirin is used in most endemic countries [17], and ethical issues about using a placebo-control group have been raised. Given the high fatality rates associated with CCHF, a well-designed multi-center, randomized controlled trial taking into account severity criteria is urgently needed in order to provide evidence-based data about ribavirin efficacy.

Recombinant and natural interferons-α exhibit activity against CCHFV *in vitro*, and might prove a promising treatment approach in the very early course of illness or as post-exposure prophylaxis, however no human studies have been conducted yet [45]. The MxA protein, an interferon-induced protein of the superfamily of large GTPases, is a key component in the interferon-induced inhibition of replication of several viruses, including CCHFV [46]. There are data suggesting that CCHFV possesses mechanisms to defeat the interferon-induced defense mechanisms by delaying IFN secretion for 48 hours post-infection [47]. This may allow the rapid progression of illness rendering the infection almost IFN-insensitive, a phenomenon that may be linked to its pathogenicity [47]. Specific laboratory findings may serve as prognostic factors for severe disease (for example severe thrombocytopenia, elevated transaminases, prolonged aPTT (activated partial thromboplastin time), decreased fibrinogen) with viral load and levels of specific cytokines playing an important factor affecting the course and the outcome of the disease [24,45,48-51]. These factors may be useful as criteria for the construction of an algorithm about whether and when antiviral medications should be given.

## Conclusions

CCHF is a disease of public health importance with a high fatality rate that has risen in incidence and displayed geographical spread over the past decade. However, to date, the driving forces behind this spread and increase in incidence remain unclear. Given this, it would be worth identifying areas at risk for CCHF and enhance surveillance. Development of new therapies and an effective and safe vaccine against CCHF would also contribute to keep CCHF under control.

## Abbreviations

CCHF: Crimean-Congo hemorrhagic fever; CCHFV: Crimean-Congo hemorrhagic fever virus; IFN: interferon.

## Competing interests

The authors declare that they have no competing interests.

## Authors' contributions

HCM conceived the review and designed the manuscript. Both authors contributed in the interpretation of literature and writing, and approved the final version.

## Funding

No funds were received.

## Pre-publication history

The pre-publication history for this paper can be accessed here:

http://www.biomedcentral.com/1741-7015/9/131/prepub
